# Computational tumor stroma reaction evaluation led to novel prognosis-associated fibrosis and molecular signature discoveries in high-grade serous ovarian carcinoma

**DOI:** 10.3389/fmed.2022.994467

**Published:** 2022-09-07

**Authors:** Jun Jiang, Burak Tekin, Lin Yuan, Sebastian Armasu, Stacey J. Winham, Ellen L. Goode, Hongfang Liu, Yajue Huang, Ruifeng Guo, Chen Wang

**Affiliations:** ^1^Department of Quantitative Health Sciences, Mayo Clinic, Rochester, MN, United States; ^2^Department of Laboratory Medicine and Pathology, Mayo Clinic, Rochester, MN, United States; ^3^Pathology Center, Shanghai General Hospital, Shanghai, China; ^4^Department of Artificial Intelligence and Informatics, Mayo Clinic, Rochester, MN, United States

**Keywords:** tumor-stroma reaction, high-grade serous ovarian carcinoma, digital pathology, prognostic fibrosis, molecular signature

## Abstract

**Background:**

As one of the key criteria to differentiate benign vs. malignant tumors in ovarian and other solid cancers, tumor-stroma reaction (TSR) is long observed by pathologists and has been found correlated with patient prognosis. However, paucity of study aims to overcome subjective bias or automate TSR evaluation for enabling association analysis to a large cohort.

**Materials and methods:**

Serving as positive and negative sets of TSR studies, H&E slides of primary tumors of high-grade serous ovarian carcinoma (HGSOC) (*n* = 291) and serous borderline ovarian tumor (SBOT) (*n* = 15) were digitally scanned. Three pathologist-defined quantification criteria were used to characterize the extents of TSR. Scores for each criterion were annotated (0/1/2 as none-low/intermediate/high) in the training set consisting of 18,265 H&E patches. Serial of deep learning (DL) models were trained to identify tumor vs. stroma regions and predict TSR scores. After cross-validation and independent validations, the trained models were generalized to the entire HGSOC cohort and correlated with clinical characteristics. In a subset of cases tumor transcriptomes were available, gene- and pathway-level association studies were conducted with TSR scores.

**Results:**

The trained models accurately identified the tumor stroma tissue regions and predicted TSR scores. Within tumor stroma interface region, TSR fibrosis scores were strongly associated with patient prognosis. Cancer signaling aberrations associated 14 KEGG pathways were also found positively correlated with TSR-fibrosis score.

**Conclusion:**

With the aid of DL, TSR evaluation could be generalized to large cohort to enable prognostic association analysis and facilitate discovering novel gene and pathways associated with disease progress.

## Introduction

Ovarian cancer (OC) is one of the leading causes of mortality among cancers in women. Pathologically, ovarian cancer is divided into high-grade and low-grade carcinomas, and the high-grade carcinomas can be further classified into various histological subtypes, most commonly serous, endometrioid and clear cell. Among them, high-grade serous ovarian carcinoma (HGSOC) is the prevalent histotype and accounts for vast majority of ovarian cancer associated mortality (1). These patients often present with rapid clinical progression, disseminated peritoneal metastasis, distant metastasis, and resistance to treatments. On the other hand, low-grade ovarian carcinomas usually present with slow-progressing diseases and are associated with much lower mortality but protracted clinical courses. Diagnostically, low-grade ovarian carcinomas can be difficult to distinguish from ovarian borderline tumors with much more indolent clinical behavior, while sometimes may share overlapping histological features with aggressive high-grade carcinomas. While all malignant OCs regardless histology types are treated similarly using platinum-based front-line chemotherapies, different surgical resections and chemotherapy treatments options could be applied to different histologic subtypes. In clinical diagnosis, recognizable histological features play a critical role in differentiating these subtypes.

Although histological diagnosis of HGSOC has been well-established, many studies have shown highly heterogenous clinical courses in these patients (2–4). Interestingly, pathologists have long observed the high variability of tumor associated stroma reaction in HGSOCs in daily practice (4, 5). Similar to a process of normal wound healing, the tumor-stroma reaction (TSR) in cancer has been associated with increased extracellular matrix and production of growth factors to facilitate recovery growth of injured tissues (6). In ovarian tumors, histopathological examination of tumor-stroma reaction is critical to differentiate low-grade serous carcinoma from serous borderline serous tumor (SBOT), with the latter lacking tumor triggered stroma reaction. More importantly, tumor-stroma reaction has been reported to facilitate tumorigenesis and associated with prognostic differences in many solid cancers such as cholangiocarcinoma, pancreatic cancer, melanoma, and OC (7–10). Though numerous studies have demonstrated that the interactions between tumor cells and stroma play a critical role in cancer progression and metastasis across multiple cancer types (11–13), the association of histological feature of stromal reaction with molecular mechanism is still underexplored. One of major reasons responsible for this gap is the lack of quantitative evaluation of TSR in solid tumors. In daily pathology practice and many research studies, TSR were examined by manually reviewing H&E-stained slides by individual pathologist, which is highly subjective and labor-intensive. Interobserver variability remains a main challenge thus limiting large-scale investigation of TSR. More importantly, evaluation of TSR by pathologist relies on personal experience, while an unbiased quantification becomes unrealistic which may cause heterogenous quality of the TSR scoring data with poorly reproducibility.

With the advancement of digital pathology, there has been substantial interest in exploring the role of quantitative attributes computationally extracted from H&E-stained whole slide images (WSI). Li et al. (14) introduced a digital pathology-based pipeline to early-stage estrogen receptor-positive invasive breast cancers for association analysis. Their results suggested that the orientation disorder of collagen fiber is prognostic for early-stage breast cancer (14). Geessink et al. (15) trained a deep learning model to segment relevant tissue types in rectal cancer histology and subsequently calculate tumor-stroma ratio for intra-tumoral stroma. Their results showed that tumor-stroma ratio is an independent prognosticator in rectal cancer when assessed automatically in user-provided stroma hot-spots (15). Failmezger et al. (16) introduced topological features extraction method to quantify stromal recruitment for immunosuppression in melanoma histology using graph based spatial model. This research revealed that tumors with high stromal clustering and barrier had reduced expression of pathways involved in naïve CD4 signaling, MAPK, and PI3K signaling, and indicated that computational histology-based stromal phenotypes within the tumor microenvironment are significantly associated with prognosis and immune exclusion in melanoma (16).

**FIGURE 1:**
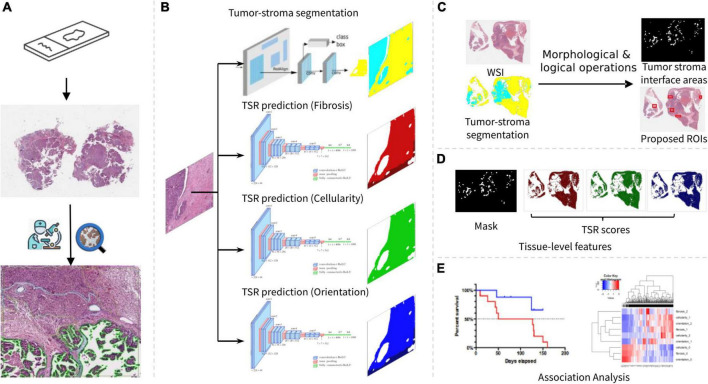
Overview of our research workflow. **(A)** Slide scanning and annotation. **(B)** Tumor-stroma segmentation and TSR score estimation. **(C)** Tumor stroma interface area identification. **(D)** Tissue-level feature summarization. **(E)** Association analysis.

In this paper, we present a digital pathology-based pipeline that is able to prognosticate patient survival by estimating degree of TSR directly from multiple aspects of digitized H&E images. Specifically, the automated pipeline consists of image processing techniques combined with several machine learning models trained from pathologists’ annotations. Serving as the cores of the pipeline, the trained models were used to identify tumor-associated stroma regions, from which we subsequently predicted TSR scores with H&E images as inputs. As shown in Figure 1, the developed pipeline was applied to our research cohort to establish associations between tissue-level features, prognosis, and molecular pathways of HGSOC.

## Materials and methods

### Cohort summary

Our research cohort consists of 291 HGSOC and 15 serous borderline ovarian tumor (SBOT) cases ascertained at the Mayo Clinic between 1994 and 2009. The SBOT cases should not have significant TSR by definition, therefore served as the negative control in both TSR score prediction and evaluation. As the cohort selection criteria, all the cases had retrievable clinical, molecular, and tissue blocks. Survival data were obtained from the Mayo Clinic Tumor Registry, electronic medical records. Gene expression profiles and histological information, including tissue sites (primary or metastasis) and tumor stage, were collected from the EHR system in Mayo Clinic. The cohort characteristics were summarized in Table 1. All the slides were scanned in Pathology Research Core at Mayo Clinic with a digital whole-slide scanner (Aperio Scanscope XT). To preserve cell and tissue details, the slides were scanned with 40× resolution (pixel size: 0.25 um). Imaging quality was manually checked by histology technicians when the slides were scanned. All patients provided informed consent for use of their tissues and data in research; all protocols were approved by the Mayo Clinic Institutional Review Board.

**TABLE 1:** Research cohort statistics.

	Overall (*N* = 291)
**Histology** *	
High grade serous	291 (100.0%)
**Age at diagnosis**	
Mean (SD)	63.337 (11.231)
Median	64.000
Q1, Q3	56.000, 71.000
Range	24.000 - 89.000
**Age at diagnosis (group)**	
[20,50] (premenopausal)	32 (11.0%)
(50,90] (postmenopausal)	259 (89.0%)
**Stage**	
3	217 (74.6%)
4	74 (25.4%)
**Grade**	
2	1 (0.3%)
3	290 (99.7%)
**Vital status at Last Follow-up**	
Alive	34 (11.7%)
Deceased	257 (88.3%)
**Months from Diagnosis to Enrollment**	
Mean (SD)	0.989 (8.425)
Median	0.000
Q1, Q3	0.000, 0.082
Range	0.000 - 107.664
**Months from Diagnosis to Last Follow-up**	
Mean (SD)	50.358 (43.148)
Median	37.072
Q1, Q3	17.763, 70.197
Range	0.263 - 196.711
**Median Time to Last Follow-up (months)**	
Events	257
Median Survival	37.434
**Debulking Status**	
Missing	1
Optimal	220 (75.9%)
Suboptimal	70 (24.1%)
Suboptimal	70 (24.1%)

*Since the SBOT cases were only included in training deep learning models for providing negative controls, the characteristics of SBOT cases were not included in this table.

### Pathologist-guided image annotation

According to the consensus of three pathologists, three types of histopathological evaluation metrics (sub-scores) were defined as criteria to characterize the extent of TSR: (i) increased fibrosis, characterized by collagen deposition, (ii) increased stromal cellularity due to fibroblastic and/or myofibroblastic proliferation, and (iii) orientation of stromal cells (14). Based on histopathologic examination of H&E-stained slide areas, the sub-TSR scores were assessed as 0 (none/weak), 1 (intermediate) or 2 (strong) (Supplementary Figure 1). The criteria were chosen to reflect the histopathologic changes commonly observed and evaluated in the clinical practice. With regard to the criterion of stromal cellularity, a minimal density of (myo)fibroblasts was assigned a score of 0, while a score of 2 was given if the area occupied by (myo)fibroblasts exceeded the area occupied by the acellular stroma in a given field. As for the fibrosis criterion, minimal deposition of fine collagen fibers with significant fiber spacing was assigned a score of 0, whereas dense collagen deposition with sclerosis was classified as 2. In terms of orientation of stromal cells, a relatively linear, unidirectional orientation was assigned a score of 2, while a haphazard orientation without appreciable directionality was scored as 0.

To create an annotated dataset for model training, five HGSOC slides were randomly selected. Within each slide, five most representative regions of interest (ROIs) were circled by pathologists in tumor-stroma interface regions, which were areas where the borders of the tumor islands came into close proximity with the surrounding stromal areas and the stroma exhibited morphologic characteristics different than the non-neoplastic ovarian stroma (Figure 1A). Two experienced pathologists were invited to annotate three TSR scores using an interactive tool named QuPath (17). In each slide, five most representative ROIs were circled by pathologists in tumor-stroma interface regions. The size of each ROI was at least 1,024*1,024 pixels. Within each ROI, polygons were used to annotate homogeneous regions with the same TSR scores. Sub-regions with the same TSR scores were labeled to the same category (Supplementary Figure 1B).

### Dataset preprocessing

Using a framework developed in our previous work (18), TSR annotations were parsed using Groovy script within QuPath and converted into a pair of image and annotation mask for each annotated ROI. For the convenience of visualization, annotation masks were encoded from dark to light R/G/B colors for each TSR scoring metric (Supplementary Figures 1D–F). To extract regular size of images and annotation masks for model training and evaluation, a 256*256 pixel sliding window was applied to the ROIs. Taking full capacity of pathologists’ annotations, the sampling stride was set to 128 for the aim of creating augmented/enlarged dataset. In total, 11,240 image patches with TSR annotations were obtained from HGSOC cases.

Considering that SBOT confirmed cases were free of significant TSR, we proposed to train the TSR prediction model with image samples from SBOT WSIs as negative controls. Thus, image patches from SBOT cases were also prepared and labeled to TSR score zero. We randomly selected five slides from our previous research (18), from which 1,405 image patches from stroma regions were randomly extracted and added into “annotated” dataset. In total, 7,025 image patches were obtained from SBOT cases.

To identify tumor-stroma interface areas and quantify TSR inside these regions, we repurposed TSR annotations for tumor-stroma segmentation modeling. The stroma region is defined as all the tissue region except the tumor region. Within each annotated ROI, overall stroma regions were obtained by merging all three different TSR score regions, while the tumor regions were defined as the remaining tissue regions inside the ROIs. The same sliding-window sampling strategy was used to extract image patches for segmentation modeling.

### Tumor-stroma tissue segmentation

In order to identify tumor-stroma interface areas where TSR occurs, tumor and stroma regions were segmented using a deep learning neural network named Mask-RCNN (19), as shown in Figure 1B. Mask -RCNN was preferred in this study as it has been used in many histological image processing tasks (20, 21) and was reported to be more robust than the U-Net for image segmentation (22, 23). The hyperparameters of Mask-RCNN, such as the dimension of convolutional layers (input dimension = 256 × 256), learning rate (lr = 0.01) and RPN anchor scales (RPN = [8, 16, 32, 64, 128]) were modified to adapt to our image segmentation task. Taking annotated dataset described in Data Preprocessing section, images from HGSOC cases were shuffled and divided into training, validation, and testing groups (3,317:1,105:1,110). From the training subset, the input layers of Mask-RCNN took both original images and tumor-stroma multilabel masks as training samples. In the training process, tumor and stroma areas were iteratively proposed by a sub-structure of Mask-RCNN named Region Proposal Network (RPN). Fully connected network layers were concatenated to the forehead layers to identify differences between ground truth (annotations) and proposed segmentation masks. By minimizing the differences, Mask-RCNN was trained to segment tumor and stroma within WSIs. To facilitate training convergence, weights from the model pretrained with Coco dataset (24) were loaded to our model as initial settings and fine-tuned on our training dataset. Specifically, the model trained at the 315th epoch reached the lowest loss in the validation set.

In the testing phase, tumor stroma regions were segmented and saved as multilabel masks for each image patch from the hold-out testing dataset using trained tumor-stroma segmentation models. Three commonly used evaluation metrics for image segmentation tasks (25, 26) were calculated to measure concordance between model prediction and ground truth, including DSC (Dice similarity coefficient), IoU (intersection of union) and AP (averaged precision).

### Tumor-stroma reaction scoring modeling

In our work, TSR score estimation was formulated into an image classification problem. In other words, different TSR scores corresponded to different image categories. We employed a commonly used DL network architecture named VGG16 (27) as our image classification model, as this model also achieved an encouraging performance on identifying tumor infiltrating lymphocytes (TIL) (28). To estimate three TSR scores, three VGG16 models (Figure 1B) were trained with the combinational dataset (from both HGSOC and SBOT cases) established in data preprocessing steps. The annotated dataset was divided into training, validation, and testing subsets (12,743:2,247:2,249, 6,000 training samples were from SBOT as negative control). During the training phase, the training dataset was divided into batches (32 samples per batch) to meet the computational resource limitations. The maximum training epoch was set to 30. At the end of each training epoch, training loss was calculated on the validation dataset. To avoid overfitting, the training process was set to stop when the loss variation is less than 10^–3^ within four epochs. To increase generalizability and avoid bias from different H&E-staining conditions, training image dataset was augmented using linear image transformation, such as rotation and flipping. With the same training strategy, three VGG16 image classification models were trained independently to estimate fibrosis, cellularity, and orientation TSR scores, respectively (Figure 1B). During the testing phase, three TSR scores were estimated for each input image from the hold-out testing dataset using the three trained VGG16 models. The performances of the three models were evaluated by comparing the concordance between model estimation and human annotation.

### Extrinsic model evaluation

Before applying our models to the entire research cohort, it was essential to evaluate model performances on an independent dataset (extrinsic) as our DL models were trained and evaluated using annotated images from ROIs (intrinsic). To this end, we developed an interactive evaluation tool (Supplementary Figure 2) for the assessment on a dataset that was independent of annotated ROIs and WSIs. For the sake of pathologists’ convenience, the original image as well as corresponding tumor-stroma segmentation and TSR scores predicted by the trained models were loaded into this evaluation tool. With the original image in the center, eight neighborhood image patches were also shown in the user interface as additional references for pathologists to make an accurate judgment. The concordance (average precision) between pathologists’ evaluations and predictions were recorded as pathologists proceeded reviewing by clicking buttons and checking boxes.

To create an independent dataset for extrinsic evaluation, image patch-level TSR score distribution was calculated for each slide. Slides with ultra-high (TSR = 2) or low (TSR = 1) TSR score ratio were selected to epitomize the performances of our trained models. Unannotated cases were selected based on TSR score ratio distribution, only the upper 5% quantile and low 5% quantile were included for manual evaluation. To limit the number of images to be evaluated, we randomly selected at least 10 but less than 30 images from each slide within tumor stroma interfacing regions.

### Applying deep learning models to research cohort

Trained DL models were generalized to the research cohort according to the following procedures.

#### Tissue detection and patch extraction

To cut down the computational cost, only the tissue regions (foreground of WSIs) were included in the testing phase. Tissue regions were detected within a down-sampled whole slide image (down-sample rate = 128), and then image patches were extracted from tissue regions accordingly. To detect foreground within down-sampled whole slide images, the color space was converted from RGB to LAB, and then threshold method was applied to L channel for tissue detections. As a commonly used foreground detection method for bright field whole slide images, feasibility of this method has been proved in our previous research (18, 29). By mapping the coordinates of pixels within detected tissue regions back to original WSI, image patches (256*256 pixels) were extracted in a tiling manner. With the same threshold-based method, foreground within the original resolution of image patches was detected. If more than 50% of the patch is background, it was excluded from our study. In the end, the extracted images were fed into our trained models for patch-level predictions.

#### Tumor-stroma interface area identification

To investigate TSR in invasive tumor front, tumor-stroma interface areas were identified based on tumor-stroma segmentation results. By down-sampling (r = 1/128) and stitching patch level results back to their original locations, slide level tissue segmentation (tumor vs. stroma) were reconstructed. Within slide level tumor-stroma segmentation results, tumor-stroma interface areas were identified using serials of image morphological and logical operations (Figure 1C and Supplementary Figure 5). The calculation process can be formulated as follows:


$$T\,u\,m\,o\,r_{c\,o\,r\,e}=C\,\left(I_{T},S\right)$$

$$S\,t\,r\,o\,m\,a_{c\,o\,r\,e}=C\,\left(I_{S},S\right)$$

$$ROI_{i\,n\,t\,e\,r\,f\,a\,c\,e}=and(xor\left[D\left(Stroma_{c\,o\,r\,e},S\right),E\left(Stroma_{c\,o\,r\,e},S\right)\right],$$

$$Stroma_{c\,o\,r\,e},D\left(Tumor_{c\,o\,r\,e},S\right)),$$

in which, I_*T*_ and I_*S*_ denotes the tumor and stroma multilabel image from slide level segmentation, respectively. S denotes the structural elements for morphological operations, including closing C(I, S), erosion E(I, S) and dilation D(I, S) (30).

To evaluate the accuracy of this automatic tumor-stroma localization method, the multilabel masks of interface areas and the counterpart WSIs were shown side-by-side and reviewed by our pathologists. Specifically, top five largest connected components were detected as the representative sub-regions for detailed reviews (31). The misidentifications were recorded for quantitative assessment metrics calculation.

#### Slide level feature summarization

To enable association analysis, TSRs were summarized to abstract slide level descriptors (Figures 1D,E). Since the ROI (tumor-stroma interface area) size varies from case to case, we used mean and standard deviation to denote slide level characteristics. Normalized distributions of TSR scores were calculated by counting TSR scores of each image patch within tumor-stroma interface regions. The entire assembled workflow was generalized to all the slides in our cohort. The summarized features were prepared for association analysis.

### Downstream association analysis

The summarized TSR characteristics were associated with clinical and molecular information. In our work, only HGSOCs were included for downstream analysis.

### Clinical associations

In HGSOC cases, for each TSR score (Fibrosis, Cellularity, and Orientation), median split was used to divide patients into two groups (i.e., score-high and low) to facilitate categorical comparisons. For univariate and multivariable [adjusted for age, FIGO stage (IV vs. III), and residual tumor after primary debulking surgery] survival analysis, a Cox proportional hazards regression model was used, and hazard ratios (HRs) and associated 95% confidence intervals (CIs) were estimated. All statistical tests were two-sided, and a *P*-value of less than 0.05 was considered statistically significant.

### Molecular associations

Tumor gene expression profiles were measured using Agilent Whole Human Genome 4x44K Expression Arrays and processed as previously described (2, 4). For gene-level association analysis, normalized expression levels of each gene were correlated with each TSR score from the same tumors using Spearman Rank correlation. For over-representation pathway analysis purposes, genes with positive and negative correlations with each TSR score (nominal *p*-value < 0.05) were analyzed using DAVID bioinformatics tool (32, 33), to reveal pathways statistically enriched in correlated gene sets. False discovery rates were computed to correct for multiple hypothesis testing.

## Results

### Tumor-stroma segmentation

**FIGURE 2:**
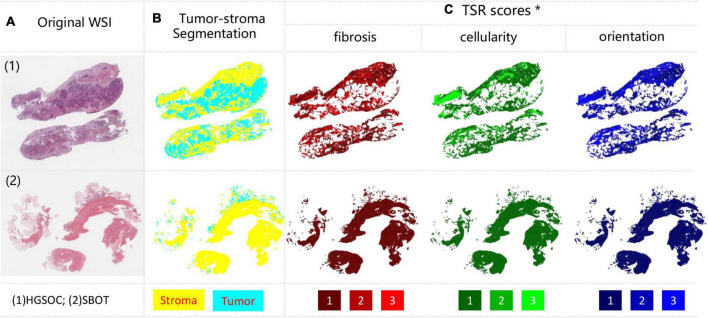
Examples of tumor-stroma segmentation and TSR scoring results. **(A)** Original WSIs, with HGSOC and SBOT each; **(B)** tumor-stroma segmentation, tumor and stroma were encoded with cyan and yellow; **(C)** TSR scores measured from three metrics, including fibrosis (Red), cellularity (Green) and orientation (Blue). Each metric was encoded from dark to light color, denoting TSR score from low to high. *For better visualization, TSR scores within all stroma regions were shown, but only the tumor-stroma interface regions were included for analysis.

The developed tumor stroma segmentation model identified the tumor vs. stroma region within both HGSOC and SBOT WSIs (Figure 2A). Figure 2B demonstrates the whole slide level segmentation results by stitching patch-level segmentation results together according to patch locations. Different tissue types were colored according to predicted categories. More examples of slide-level segmentations were shown in Supplementary Figure 3.

**FIGURE 3:**
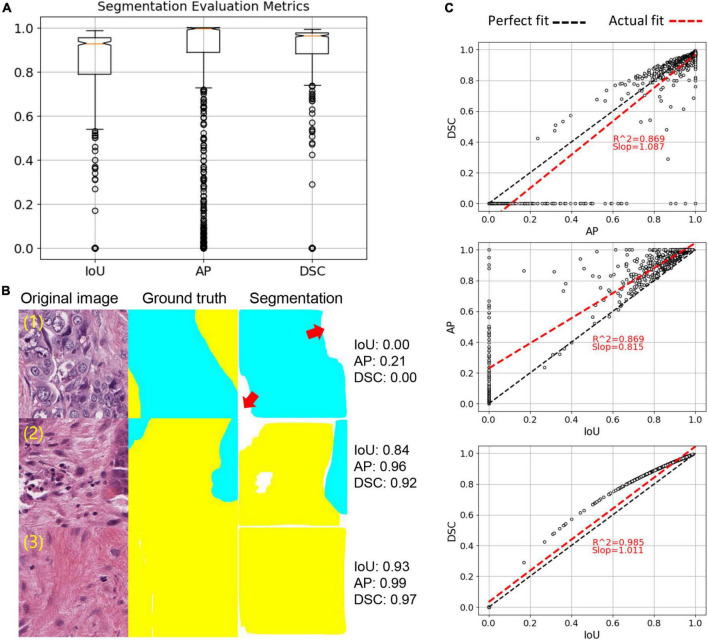
Tumor-stroma segmentation evaluation. **(A)** Boxplot of three evaluation metrics, including IoU, AP and DSC. **(B)** Examples of segmentation. Red arrows point to missed targets in segmentation. **(C)** Correlation of three evaluation metrics. Each dot represents an image sample. Linear regression was used to calculate correlation.

In the hold-out testing subset (*N* = 1,110) reserved for segmentation accuracy assessment, DSC, IoU and AP achieved 93.5, 88.65, and 95.34%, respectively (Figure 3A). Moreover, we observed that IoU and DSC dramatically decreased if there was a tissue type misdetection (Figure 3B). Our evaluation also suggested that IoU and DSC were highly correlated to each other (Figure 3C), and AP could be a more suitable metric for measuring our segmentation accuracy. Since the hold-out testing set is from annotated ROIs, our evaluation results suggested that the trained tumor-stroma segmentation model performed well in intrinsic cases.

Based on the criteria mentioned in the methods, 15 unannotated slides were identified, from which 615 image patches were sampled for independent tumor-stroma segmentation and TSR score evaluation. By analyzing the review records from the extrinsic evaluation tool (Supplementary Figure 2), our model achieved 90.6% accuracy, indicating that pathologists generally agreed with our tumor-stroma segmentation performance within independent WSIs. It is noteworthy that our trained model can be applied to the entire research cohort to generate tumor-stroma segmentation across the whole slide for the downstream analytical steps.

Based on our tumor-stroma segmentation results, some WSIs with low stroma tissue areas were identified. By checking the original images of these cases within QuPath, the pathologists confirmed that our tumor-stroma segmentation results were accurate, as the tumor islands occupied the majority of these slides, while the stromal areas consisted of a significant number of adipocytes, necrosis, and/or hemorrhage, with minimal collagenous and cellular stroma [Supplementary Figure 4 case (4)].

### Tumor-stroma reaction scoring and evaluations

Using our trained models, three TSR scores were predicted for each patch inside the stroma regions based on the tumor stoma segmentation results. The spatial overview of slide-level TSR was reconstructed by mapping three TSR scores to different colors with ranked saturation (Figure 2C). More examples of slide level segmentations are shown in Supplementary Figure 4. More zoomed in results can be found in our GitHub repository.

**FIGURE 4:**
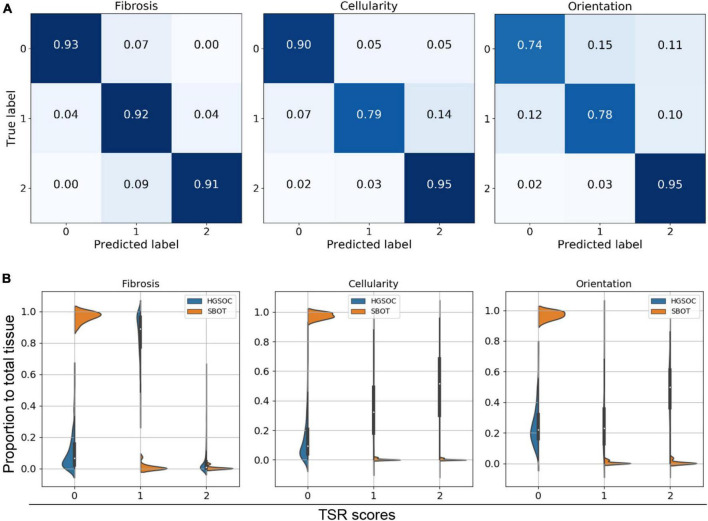
Tumor-stroma reaction (TSR) scoring evaluation. **(A)** Confusion matrix of intrinsic evaluation. **(B)** Violine plot for three TSR metrics within HGSOC vs. SBOT. Majority of SBOT images have low TSR score, no matter in which metric.

Our qualitative results suggested that the predicted TSR scores were not even in stroma regions of all HGSOC. Heterogeneity between regions and cases were high, as shown in Figure 2C and Supplementary Figure 4. Confusion matrices were calculated to quantitatively evaluate model performances on the hold-out testing dataset (*N* = 2,249). The results indicated that our model achieved accurate TSR score estimation, especially in predicting fibrosis (>90%), as shown in Figure 4A. In the extrinsic evaluation, with an average precision over 82.8%, the results suggested that the trained model can be generalized to our research cohort for objective TSR scoring.

We also observed false positives (TSR scores > 0) in some region from three SBOT cases, as illustrated Supplementary Figure 4 case (3). By mapping the TSR scores back to the WSIs and observing with high resolution, we identified that these false-positive predictions were presumably due to these slides having mostly non-neoplastic ovarian stroma, which inherently has a relatively cellular and fibrotic composition. Since our model is mainly trained on annotated HGSOC regions, the trained model did not capture texture patterns within normal SBOT regions.

Violin plots illustrated the distributions of TSR score ratios per case. Figure 4B indicates that the majority of image patches had low TSR scores (TSR = 0), regardless of the diagnosis being SBOT or HGSOC. However, compared to SBOT cases, HGSOC cases were more likely to have higher TSR scores (>1), especially for fibrosis score. We observed a significant proportion of image patches from HGSOC cases had fibrosis TSR scores of 1. After checking the training dataset, we confirmed that half (3,339 out of 6,743) of the annotated images of HGSOC cases had moderate fibrosis TSR scores, indicating that the ambiguity of fibrosis scores could be high, and our TSR scoring model was trained to match pathologists’ interpretations.

### Identification of tumor-stroma interface regions

Our tumor-stroma interface region identification strategy identified five regions within each testing slide (Supplementary Figure 5). The proposed interface regions were localized and overlayed to the WSIs. According to pathologists’ manual review, 83.4% (207 out of 250) proposed tumor-stroma regions were confirmed to be tumor-stroma interface area. After checking the falsely proposed tumor-stroma interface regions, we found flaws of tumor-stroma segmentation in those regions to be responsible for the failure, indicating that the tumor-stroma interface identification relies heavily on tumor-stroma segmentation results. To facilitate replication of our work, all the code for this paper is public available on GitHub.^1^ The pretrained models for tumor-stroma segmentation and tumor-stroma reaction prediction can be obtained *via* contacting authors.

### Tumor-stroma reaction clinical and molecular associations

**FIGURE 5:**
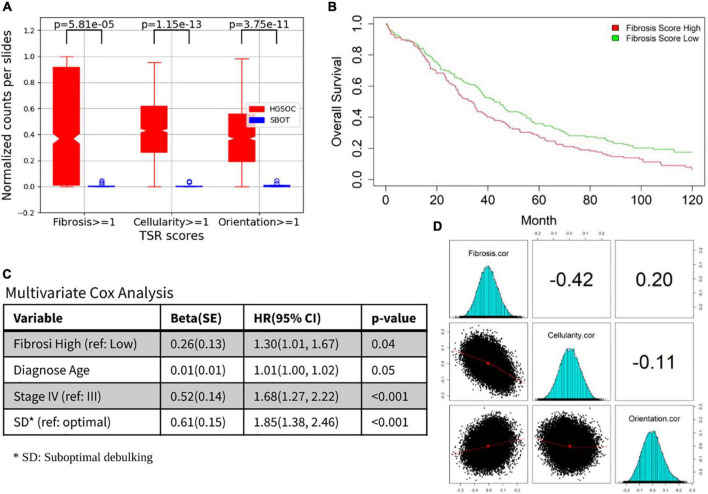
Prognosis and molecular associations of fibrosis score. **(A)** Tumor-stroma reaction (TSR) score boxplots for HGSOC and SBOT groups. **(B)** Overall survival differences between fibrosis high vs. low. **(C)** The prognostic association for other established prognostic factors (age at diagnosis, stage and residual tumor after surgical debulking). **(D)** Correlation between fibrosis/cellularity/orientation and molecular findings.

All three TSR scores were significantly elevated in HGSOC cases vs. SBOT cases (*p* < 0.001, Figure 5A). Moreover, in HGSOC cases, higher fibrosis score (>median) was significantly associated with worse survival (*p* = 0.02; Figure 5B), and the prognostic association remained significant (*p* = 0.04; Figure 5C) after multivariate adjusting for other established prognostic factors (age at diagnosis, stage, and residual tumor after surgical debulking). In order to gain further insight into possible molecular mechanisms associated with each TSR score, gene-level correlations were computed between mRNA level of each gene and TSR score from the same tumors; and significant associations were found in two correlations: (1) correlation between fibrosis and molecular findings, and (2) correlation between cellularity and molecular findings (Figure 5D). Further genetic analysis suggested different molecular bases between the three TSR scores. Through pathway enrichment analysis, genes positively correlated with TSR-fibrosis score were found to be enriched in 14 KEGG pathways [FDR (false discovery rate) < 5%], which are mostly associated with cancer signaling aberrations. On the other hand, genes positively correlated with the TSR-orientation score were enriched in 79 KEGG pathways, with leading significant pathways implicated with immune response (Supplementary Table 1). In contrast, genes having positive correlations with TSR-cellularity score were only significantly enriched with one KEGG pathway (hsa01100: Metabolic pathways; FDR = 0.04). Detailed molecular association results including gene- and pathway-level results were shown in Supplementary Table 1.

## Discussion

In this study focusing on digital analysis of reactions between tumor and stroma, combined with a critical pathology review using subjective scoring systems, we demonstrated highly concordant computational prediction based on VGG16 DL structure with training annotations and independent validations by multiple pathologists in HGSOC. Conventionally, TSR is often lumped as an overall subjective assessment by pathologists. Herein we further dissected it into three essential aspects of TSR and examined their individual and combined significance by digital detection. With a series of digital detection and quantification procedures including tumor-stroma interface area detections, the trained DL model has been successfully generalized to a large OC cohort from a single institution consisting of nearly 300 patients with long-term clinical follow-up and tumor transcriptome data. Interestingly, among the three aspects of TSR, the data revealed significant prognosis association only with fibrosis score. This is the first study demonstrating the outstanding significance of fibrosis over other TSR pathological features in HGSOC, indicating these features did not carry the equal weight regarding clinical significance. At the design phase of this study, the orientation score was selected as one of the three quantification criteria, mainly to further characterize the fibroblastic and/or myofibroblastic proliferation. In fact, collagen fiber organization has been associated with prognosis in breast cancer in the literature and DL approaches have been employed to quantify this as a histomorphometric feature (14, 34). However, our team observed this criterion to be a highly subjective one. In addition, the orientation score did not reveal any significant prognosis associations in our transcriptome association analysis, calling the usefulness of this criterion with regard to ovarian cancer TSR assessment into question. These observations warrant further study on individual components of the TSR, as well as aberrant gene- and pathway-level activities associated with different digital TSR scores.

Of note, the design of the TSR scoring in our study was not specific or limited to HGSOC, and the same histological principle can be applied to in majority types of solid cancers. Therefore, the digital platform developed by our study can be potentially generalized and applied to various tumor types. These findings highlight potentials of powerful DL approaches to generalize digital pathology-based predictions for large-scale translational research and enable molecular discoveries to better understand tumorigenesis and cancer progression.

To consolidate our discoveries, we considered including other pre-existing cases into our research cohort. However, we found that images scanned at different times could be dramatically different in hue even if they were from the same patient, same institution (Mayo Clinic) and shared the same staining and image acquisition protocol. This inconsistency may be due to multiple technical variables, including scanner settings and/or age of the H&E-stained slides. Since these batch effects in pathology image data could be hidden variables in deep learning digital pathology that compromise the accuracy of classification systems (35, 36), we opted for not including our previous HGSOC data into this research. Many previous studies introduced color normalization methods to minimize staining inconsistencies (29, 37). Though it is hard to measure the preservation of diagnostic information after image transformation, many integrative studies investigating cancer subtype classification and prognosis association achieved optimistic performances by introducing image normalization (38, 39). From this point of view, color normalization could be beneficial for assorted research cohort from miscellaneous data sources, especially from multiple institutions. Meanwhile, we also noticed that some investigators improved their model performance by synthesizing images using generative adversarial network (GAN) (40, 41), which could be another potential way to enhance the generalizability of our TSR estimation model.

Although our tumor-stroma interface region detection relies on patch-level tumor-stroma segmentation, the strategy we introduced (Supplementary Figure 5) could partially offset this limitation. To generate a slide-level overview of tissue context (tumor vs. stroma) for image morphological manipulations, patch-level tumor-stroma segmentation results were down-sampled and stitched back to their original locations. In this process, the tissue type (tumor or stroma) in slide-level was determined by the dominated component of patch-level segmentation. In other words, for tumor-stroma interface region detection task, tumor-stroma segmentation results were not required to achieve pixel-level accuracy.

We observed that our TSR scoring models highlighted some regions with a potentially high TSR in five SBOT cases, for example, case (3) in Supplementary Figure 4. The main reason contributing to this flaw is that our models were not trained to differentiate normal vs. abnormal ovarian stroma. We anticipate that our TSR scoring pipeline can achieve a better estimation if models can be trained using extra normal vs. abnormal ovarian stroma annotations. Meanwhile, we acknowledge that using TSR as the sole measurement is not enough to describe the complex tumor micro-environment (TME). It has been reported that TILs can also be assessed with the aid of digital pathology in advanced-stage, HPV-negative head and neck tumors (42). We will introduce more interpretable measurements for pathology image metadata summarization, which will bring more opportunities for novel discoveries. To integrate cellular level features into large cohort analysis, we also plan to introduce more advanced cell segmentation modules to our workflow for better cell level representations (43). As reported in our previous work (44), over- and under-segmentations lead to inaccurate downstream analysis impute to erroneous features calculated based on them.

Another imperfection of our study is way we summarize predicted TSR score to slide level for association analysis. For the sake of simplicity, we employed simple statistics and assigned equal weights to patch level TSR predictions. However, the relative importance of tissue regions contributing to the diagnosis could be dramatically different depending on tissue context. In this study, we introduced tumor-stroma interface area identification methods that were aimed at mimicking pathologists’ diagnoses. This strategy is simple and works well in most cases; however, it highly depends on the tumor-stroma segmentation accuracy. We noticed that some studies proposed to introduce multi-resolution analysis for capturing subtle tissue features within different WSI scales (45, 46). Attention based deep neural networks (47) are also tangible options for locating diagnostically relevant regions and assign those regions with higher weights in automatic analysis. We will consider these solutions to fill the gaps in our current work.

Further limitations of the current study include that all the samples were from a single institution, requiring further validations in external cohorts. H&E imaging-based digital pathology studies as such may be also affected by paraffin block preservation protocols and digital scanning parameter settings, which could lead to model differences when generalizing to WSI samples collected and scanned following different protocols. Computational developments and evaluations will be made to address these challenges.

## Conclusion

Our developed system achieved encouraging performances in tissue segmentation and TSR score predictions and generalized successfully to a large single-institution OC cohort, resulting in novel discoveries of clinical prognosis associations and molecular findings implicated in different TSR scores.

## Data availability statement

The original contributions presented in this study are publicly available. The source code can be found here: https://github.com/smujiang/TumorStromaReaction. The pre-trained model can be obtained by contacting authors.

## Ethics statement

The studies involving human participants were reviewed and approved by Mayo Clinic Institutional Review Board. The patients/participants provided their written informed consent to participate in this study. Written informed consent was not obtained from the individual(s) for the publication of any potentially identifiable images or data included in this article.

## Author contributions

RG and YH conceived of the presented histological concept. BT, LY, and RG defined data annotation criteria, performed annotation, and validated model performance. JJ and CW conceived and performed majority of experiments and manuscript drafting. SA conducted cohort characteristic statistics. SW contributed to the final version of the manuscript. HL provided funding resources and supervised the deep learning parts of this project. EG also supervised the funding of this work. All authors discussed the results and contributed to the final manuscript.

## Footnotes

1
https://github.com/smujiang/TumorStromaReaction

## Supplementary material

Supplementary Figure 1 Tumor-stroma reaction (TSR) annotation. **(A)** Original image within ROI selected for annotation. **(B)** Annotated ROIs. Polygons were used to label regions with different TSR scores. **(C)** Legend of three TSR score measurements. **(D–F)** Parsed annotations. TSR scores were encoded into R/G/B colors to represent three measurements (fibrosis, cellularity, and orientation), respectively; Panels **(G,H)** are two zoom in examples. Panel **(G)** was annotated as Fibrosis = 2, Cellularity = 1, Orientation = 1; Panel **(H)** was annotated as Fibrosis = 1, Cellularity = 2, Orientation = 2.

Supplementary Figure 2 Interactive tool for extrinsic evaluation. Source code available in our GitHub. Buttons and checkboxes on the right are clickable, pathologists’ interactions were recorded for extrinsic evaluation.

Supplementary Figure 3 Extra examples of tumor-stroma segmentation results, including five HGSOCs, five SBOTs and their tumor stroma segmentation results.

Supplementary Figure 4 Extra examples (two HGSOC and two SBOT) of TSR scoring results. TSR scores measured with fibrosis (Red), cellularity (Green), and orientation (Blue). From dark to light, TSR scores were encoded into R/G/B colors. *For better visualization, TSR scores within all stroma regions were shown, but only the tumor-stroma interface regions were included for analysis.

Supplementary Figure 5 Tumor-stroma interface area identification. **(A)** Original WSI and tumor-stroma segmentation results. **(B)** Morphological and logical operations were conducted on tumor-stroma segmentation for localizing tumor-stroma interface regions. **(C)** Proposed ROIs (red rectangles) for TSR score summarization.

Supplementary Table 1 Detailed molecular association results, including top 10 genes positively/negatively associated with TSR-Fibrosis score, and pathways positively associated with Fibrosis and Orientation scores, respectively.
