# Supplementary figures and images for: Computational tumor stroma reaction evaluation led to novel prognosis-associated fibrosis and molecular signature discoveries in high-grade serous ovarian carcinoma
Source: Front Med (Lausanne). 2022 Sep 7;9:994467. doi: 10.3389/fmed.2022.994467 (PMC9490262; doi:10.3389/fmed.2022.994467)

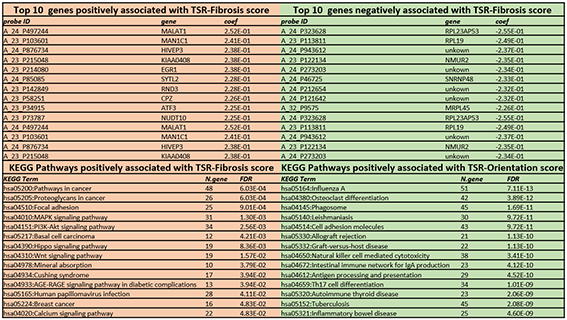

Supplement: Supplementary file 1 [file Image_1.TIF]

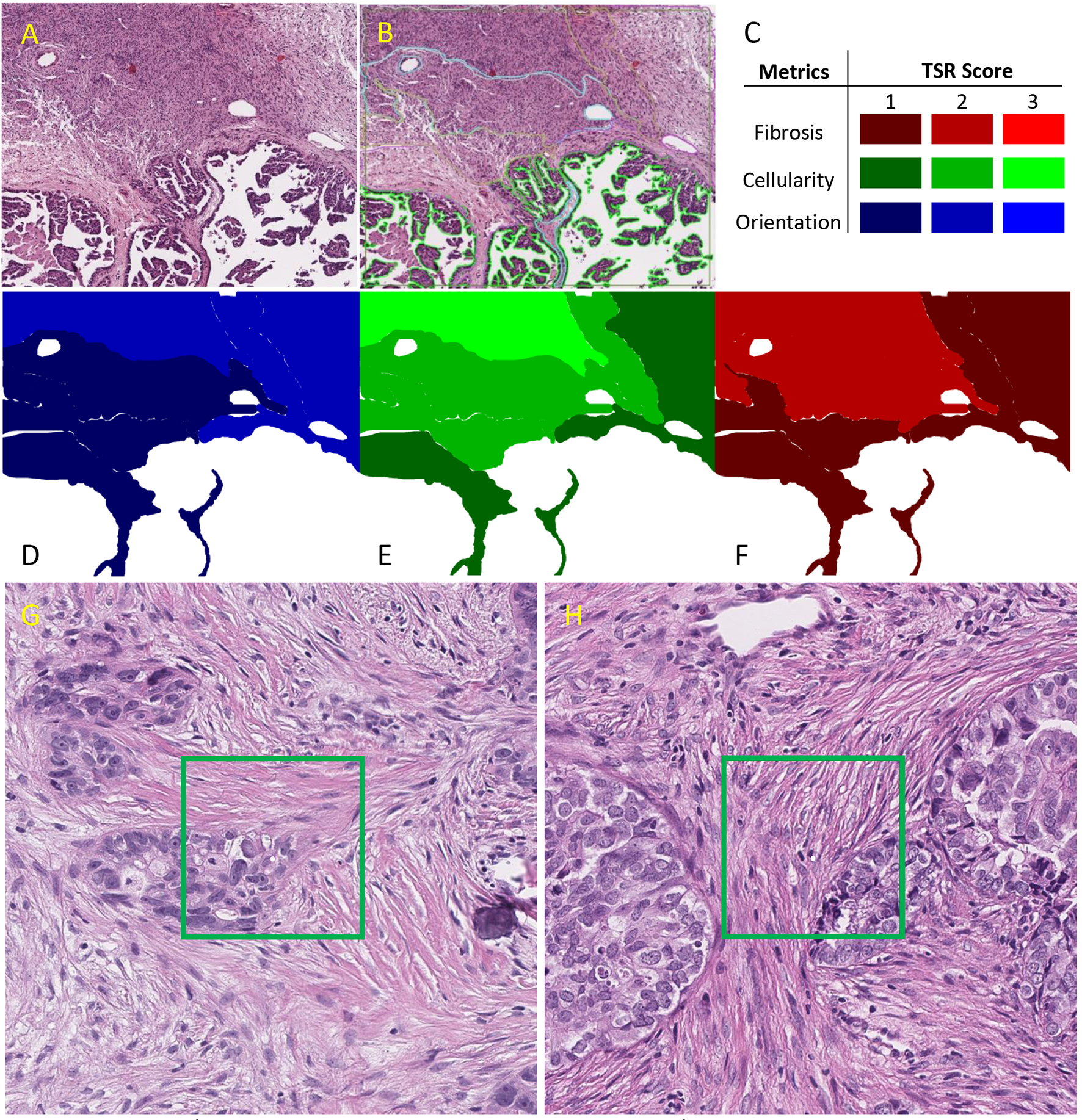

Supplement: Supplementary Figure 1 — Tumor-stroma reaction (TSR) annotation. (A) Original image within ROI selected for annotation. (B) Annotated ROIs. Polygons were used to label regions with different TSR scores. (C) Legend of three TSR score measurements. (D–F) Parsed annotations. TSR scores were encoded into R/G/B colors to represent three measurements (fibrosis, cellularity, and orientation), respectively; Panels (G,H) are two zoom in examples. Panel (G) was annotated as Fibrosis = 2, Cellularity = 1, Orientation = 1; Panel (H) was annotated as Fibrosis = 1, Cellularity = 2, Orientation = 2. [file Image_2.TIF]

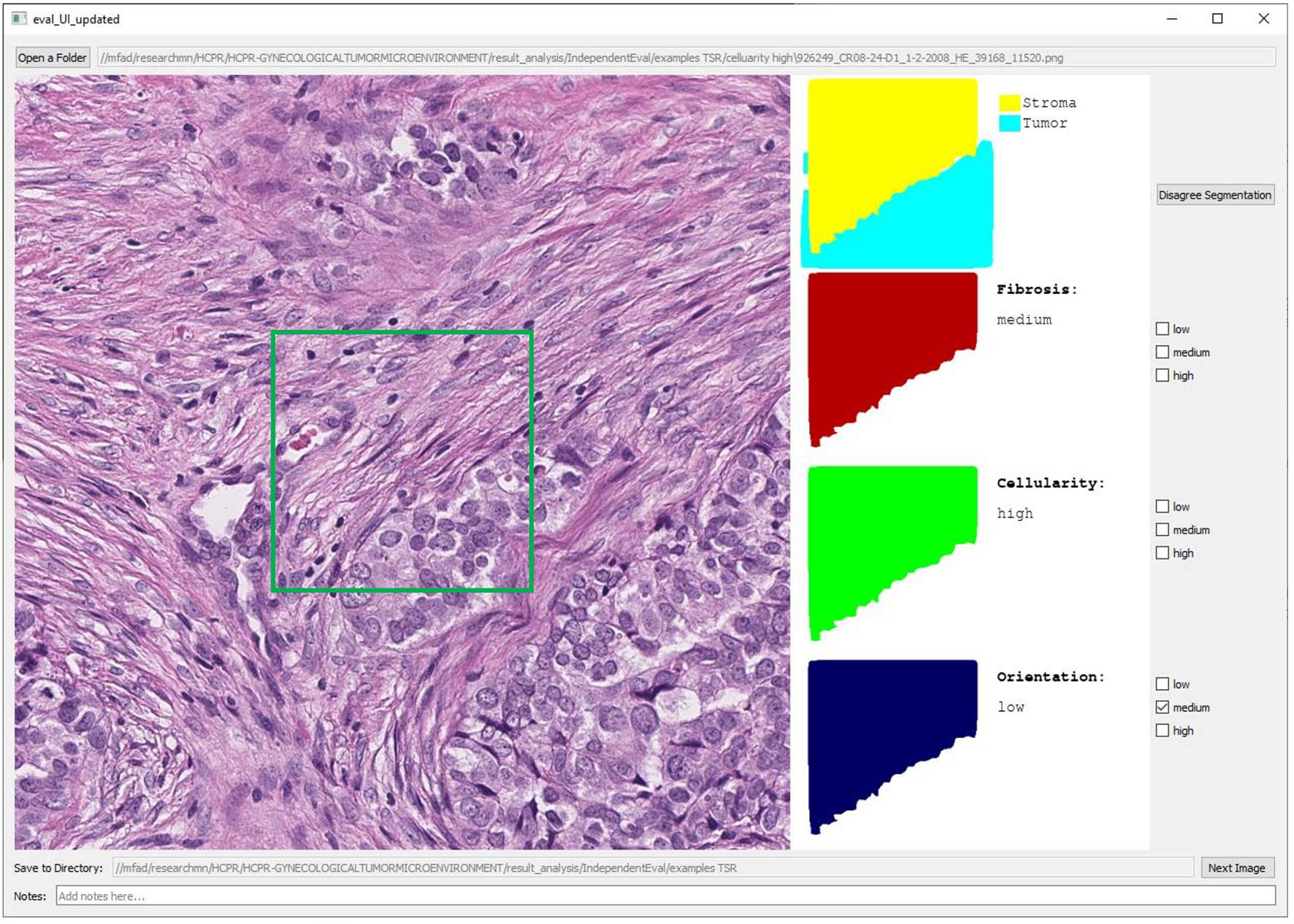

Supplement: Supplementary Figure 2 — Interactive tool for extrinsic evaluation. Source code available in our GitHub. Buttons and checkboxes on the right are clickable, pathologists’ interactions were recorded for extrinsic evaluation. [file Image_3.TIF]

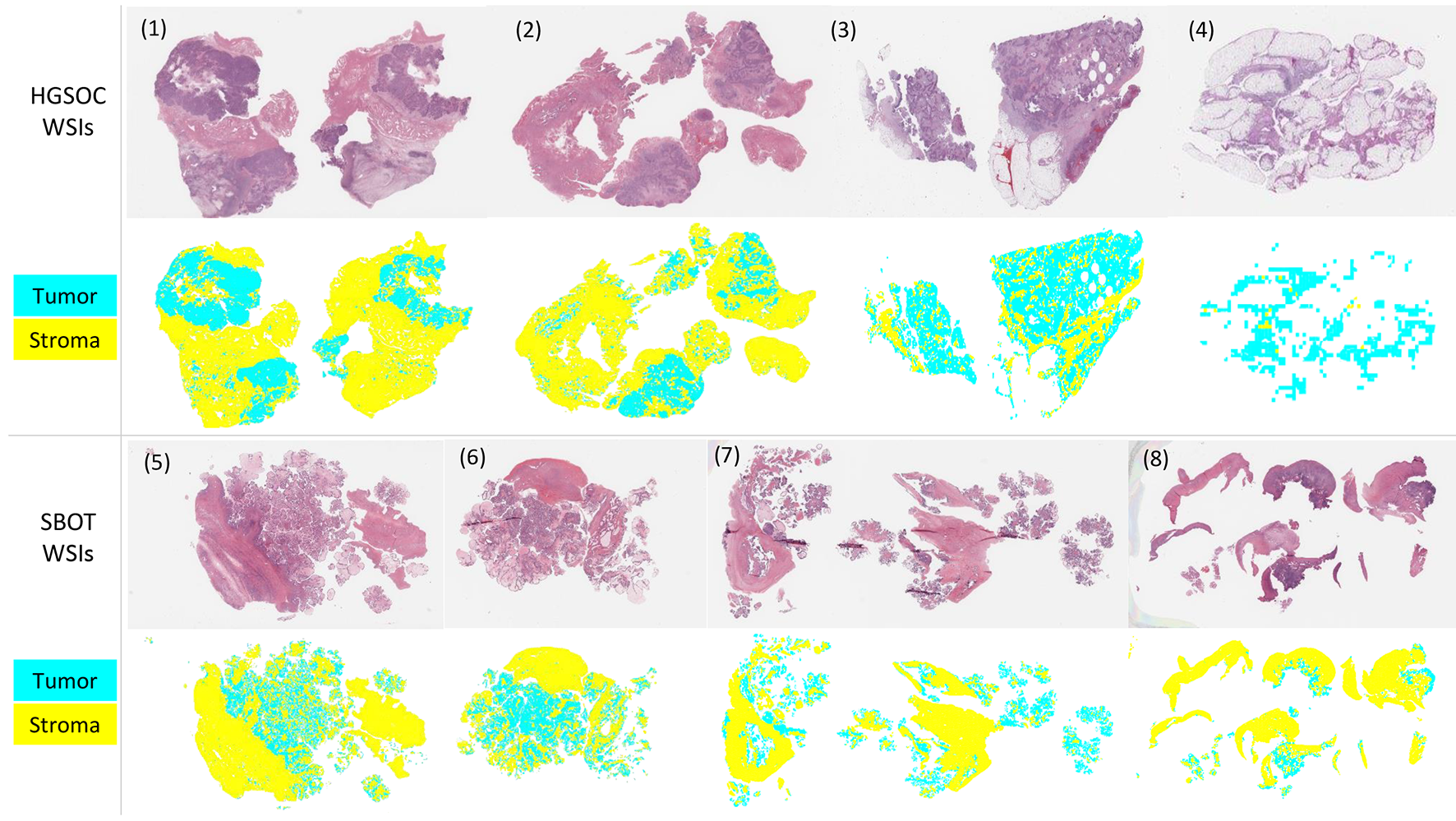

Supplement: Supplementary Figure 3 — Extra examples of tumor-stroma segmentation results, including five HGSOCs, five SBOTs and their tumor stroma segmentation results. [file Image_4.TIF]

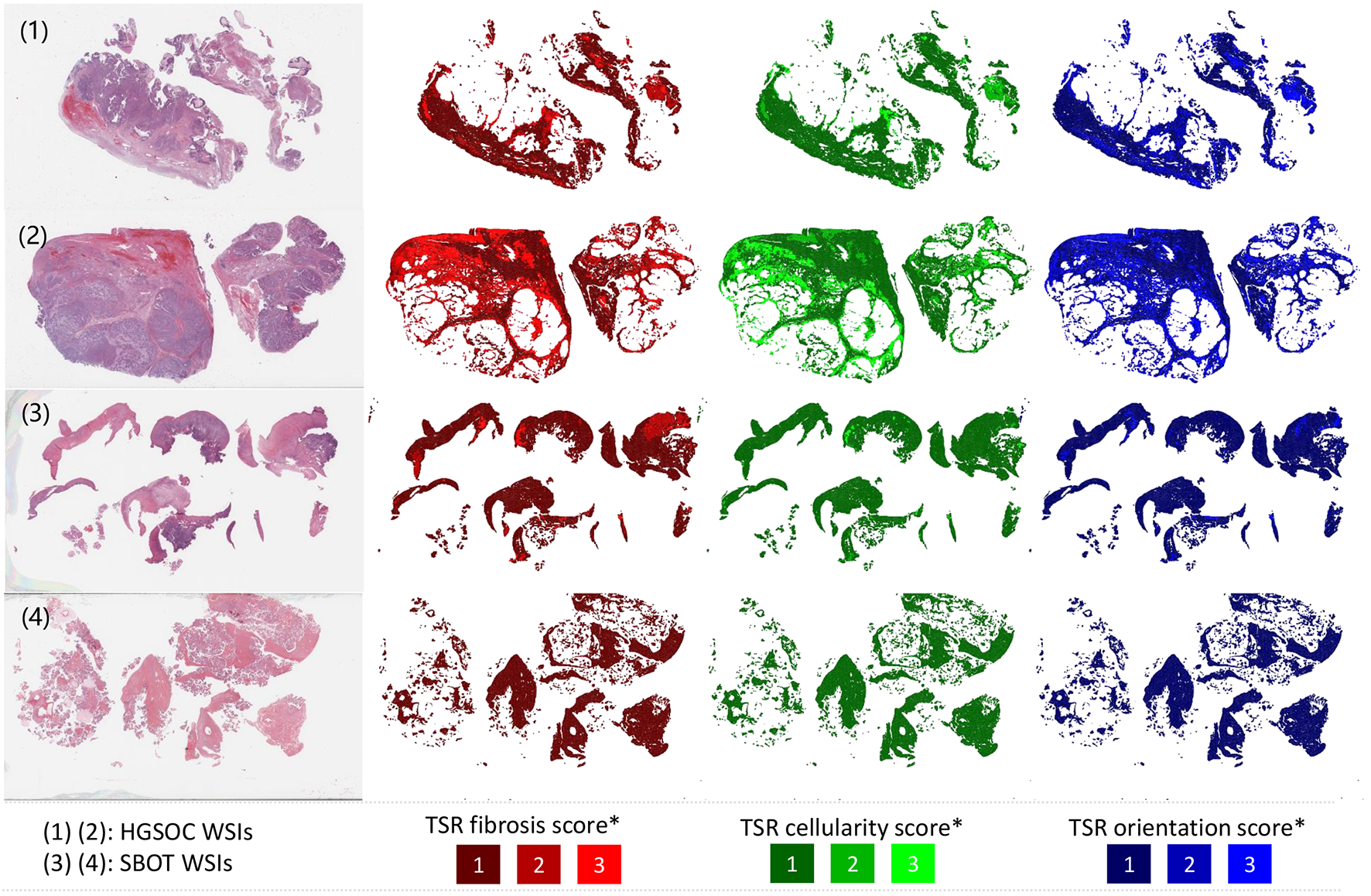

Supplement: Supplementary Figure 4 — Extra examples (two HGSOC and two SBOT) of TSR scoring results. TSR scores measured with fibrosis (Red), cellularity (Green), and orientation (Blue). From dark to light, TSR scores were encoded into R/G/B colors. *For better visualization, TSR scores within all stroma regions were shown, but only the tumor-stroma interface regions were included for analysis. [file Image_5.TIF]

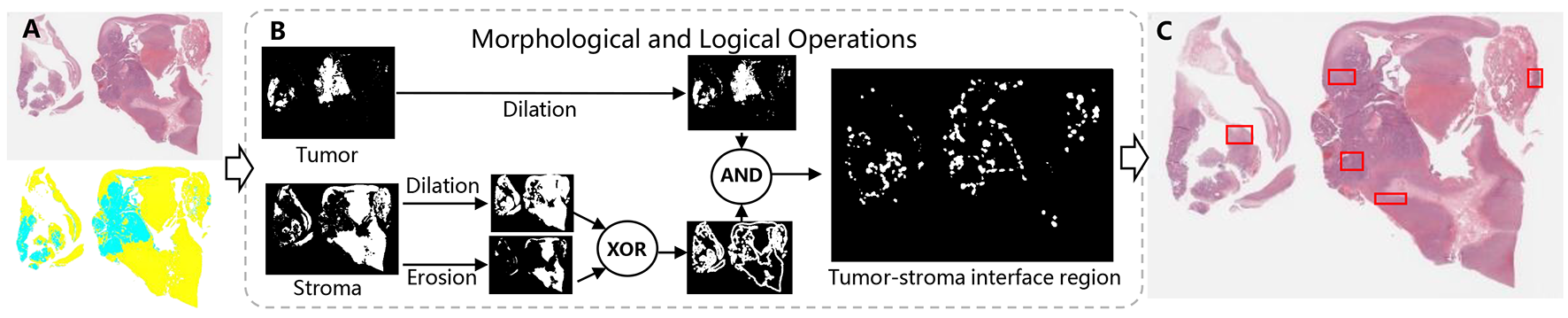

Supplement: Supplementary Figure 5 — Tumor-stroma interface area identification. (A) Original WSI and tumor-stroma segmentation results. (B) Morphological and logical operations were conducted on tumor-stroma segmentation for localizing tumor-stroma interface regions. (C) Proposed ROIs (red rectangles) for TSR score summarization. [file Image_6.TIF]
