# Supplementary material for: Computational tumor stroma reaction evaluation led to novel prognosis-associated fibrosis and molecular signature discoveries in high-grade serous ovarian carcinoma
Source: Front Med (Lausanne). 2022 Sep 7;9:994467. doi: 10.3389/fmed.2022.994467 (PMC9490262; doi:10.3389/fmed.2022.994467)
Supplement: Supplementary Table 1 — Detailed molecular association results, including top 10 genes positively/negatively associated with TSR-Fibrosis score, and pathways positively associated with Fibrosis and Orientation scores, respectively. [file Data_Sheet_1.PDF]

Table 1. Research cohort statistics

	Overall (N=291)
**Histology***	
High grade serous	291 (100.0%)
**Age at diagnosis**	
Mean (SD)	63.337 (11.231)
Median	64.000
Q1, Q3	56.000, 71.000
Range	24.000 - 89.000
**Age at diagnosis (group)**	
[20,50] (premenopausal)	32 (11.0%)
(50,90] (postmenopausal)	259 (89.0%)
**Stage**	
3	217 (74.6%)
4	74 (25.4%)
**Grade**	
2	1 (0.3%)
3	290 (99.7%)
**Vital status at Last Follow-up**	
Alive	34 (11.7%)
Deceased	257 (88.3%)
**Months from Diagnosis to Enrollment**	
Mean (SD)	0.989 (8.425)
Median	0.000
Q1, Q3	0.000, 0.082
Range	0.000 - 107.664
**Months from Diagnosis to Last Follow-up**	
Mean (SD)	50.358 (43.148)
Median	37.072
Q1, Q3	17.763, 70.197
Range	0.263 - 196.711
**Median Time to Last Follow-up (months)**	
Events	257
Median Survival	37.434
**Debulking Status**	
Missing	1
Optimal	220 (75.9%)
Suboptimal	70 (24.1%)

*Since the SBOT cases were only included in training deep learning models for providing negative controls, the characteristics of SBOT cases were not included in this table.
